# Treatment for Nontype 1 Retinopathy of Prematurity by Intravitreal Injection of Antivascular Endothelial Growth Factor Drugs

**DOI:** 10.1155/2022/6266528

**Published:** 2022-11-21

**Authors:** Haitao Zhang, Xin Yang, Fangfang Zheng, Xiangke Yin, Suhua Wan

**Affiliations:** ^1^Henan Provincial People's Hospital, Henan Eye Hospital, Henan Eye Institute, People's Hospital of Zhengzhou University, Zhengzhou 450003, China; ^2^Neonatal Intensive Care Unit of Henan Provincial People's Hospital, People's Hospital of Zhengzhou University, Zhengzhou 450003, China

## Abstract

**Background:**

To explore clinical characteristics and treatment reasons for intravitreal injection of antivascular endothelial growth factor (anti-VEGF) drugs in the treatment of nontype 1 retinopathy of prematurity (ROP).

**Methods:**

A retrospective study was conducted to screen the nontype 1 ROP from the collected ROP patients who received intravitreal injections of anti-VEGF drugs in Henan Eye Hospital from September 2018 to June 2021.

**Results:**

A total of 138 ROP cases (262 eyes) were included in this study, including 39 cases (28.3%), 65 eyes (24.8%) that were the nontype 1 ROP. Compared with the type 1 ROP group, the nontype 1 ROP group had slightly later treatment time (39.8 ± 2.7 weeks vs 38.1 ± 2.6 weeks, *P* < 0.05) and a higher proportion of fusion protein drugs (87.2% vs 54.5%, *P* < 0.05). After intravitreal injection of anti-VEGF drugs, 27 eyes (41.5%) were cured and 38 eyes (58.5%) improved in the nontype 1 ROP group, without recurrence and aggravation cases. There were more lesions in zone II (63 eyes, 96.9%), with stage 2 (40 eyes, 61.5%) and stage 3 (23 eyes, 35.4%), and 58 eyes (89.2%) showed preplus in the nontype 1 ROP group. Treatment reasons included preplus in 58 eyes (89.2%), ridge aggravation in 22 eyes (33.8%), simultaneous treatment of the contralateral eye in 9 eyes (13.8%), no regression of lesions in the persistent stage 2 or 3 over PMA 44 weeks of follow-up in 8 eyes (12.3%), and logistical considerations in 4 eyes (6.2%).

**Conclusions:**

Considering some peculiar clinical characteristics, treatment by intravitreal injection of anti-VEGF drugs may be considered carefully for some nontype 1 ROP in critical conditions.

## 1. Introduction

Retinopathy of prematurity (ROP) is a proliferative retinal vascular disease that occurs in premature and low-weight infants, and it mostly afflicts both eyes and may cause retinal detachment and blindness. Current treatments of ROP include laser photocoagulation and intravitreal injection of antivascular endothelial growth factor (anti-VEGF) drugs. The Early Treatment for Retinopathy of Prematurity (ETROP) and the latest expert consensus or guidelines recommend treatment for type 1 ROP and close follow-up for type 2 ROP [1, 2]. However, some ROP eyes have special manifestations in clinical practices, although they have not reached the level of type 1 ROP, manifested as gradually thickened or widened ridges without typical manifestation of plus disease or no regression of the retinal lesions for a long time of follow-up. This kind of ROP is more serious than type 2 ROP, and it is difficult to decide whether to continue follow-up or give treatment. These nontype 1 ROP eyes may be recommended for treatment to avoid unnecessary medical disputes or irreversible vision damage caused by the aggravation of the disease.

Previous studies have reported that 9–27% affected eyes of patients with nontype 1 ROP receive treatment in different countries [3–6]. In recent years, intravitreal injection of anti-VEGF drugs for the treatment of ROP, with advantages of simple operation, minimal invasion, and continuous growing of retinal vessels to the periphery after injection, has gradually become an important treatment method [7–9]. There is a lack of relevant research on the treatment of intravitreal injection of anti-VEGF drugs for nontype 1 ROP in critical conditions. In this study, the nontype 1 ROP cases who had received intravitreal injection of anti-VEGF drugs in our hospital were enrolled to analyze the treatment effect, clinical characteristics, and treatment reasons and to explore the personalized diagnosis and treatment for ROP with special clinical characteristics.

## 2. Methods

### 2.1. Subjects and Data

This was a retrospective study. ROP infants who had received intravitreal injection of anti-VEGF drugs were collected in the Department of Ophthalmology of Henan People's Hospital (Henan Eye Hospital) from September 2018 to June 2021, of whom, type 1 and nontype 1 ROP cases were subgrouped. This study was conducted with the approval of the Medical Ethics Committee of Henan Eye Hospital (Approval Number HNEECKY-2021 (49)) and performed in accordance with the Declaration of Helsinki. All methods were confirmed to be performed with relevant regulations. All infant's parents or legal guardians signed the written informed consent prior to treatment.

### 2.2. Inclusion and Exclusion Criteria

Diagnostic criteria for type 1 ROP were as follows [1]: (1) ROP at stages 1–3 with plus in zone I; and ROP at stage 3 without plus in zone I; (2) stage 2 or 3 ROP with plus in zone II; (3) aggressive ROP (A-ROP). Those who did not meet any above conditions and received intravitreal injection of anti-VEGF drugs were defined as nontype 1 ROP. Infants with unstable vital signs caused by systemic diseases in the heart, brain, lung, or accompanied by other fundus lesions were excluded.

### 2.3. Study Selection

The retinal examination of ROP infants was performed in the neonatal intensive care unit (NICU) and ophthalmic clinic by two experienced doctors under topical anesthesia after mydriasis with RetCam 3. All abnormal retinal images were judged by a senior pediatric retinal professor (Haitao Zhang, associated professor), and then, diagnosis and treatment suggestions were made. Information of name, gender, gestational age (GA), birth weight (BW), and the time of examination and injection were recorded after the retinal examination. The zone, stage, range, and plus disease of binocular retinal lesions were recorded according to ICROP3 [10].

Anti-VEGF drugs used in the study were ranibizumab (0.25 mg/0.025 ml), conbercept (0.25 mg/0.025 ml), and aflibercept (1 mg/0.025 ml). The latter two drugs were fusion proteins. Intravitreal injection for ROP was performed under topical anesthesia. After local disinfection, a 29G needle-equipped syringe was used to penetrate the eyeball wall at 1.0–1.5 mm posterior of the limbus to the vitreous cavity parallel to the optical axis. Antibiotic eye drops were used for 3–5 days to prevent ocular infection, and the first eye review was conducted within 7 days. The interval of the next review (1–3 weeks) should be determined according to the retinal manifestations, and patients should be followed up until complete retinal vascularization or for at least 24 weeks.

The characteristics of included subjects were analyzed according to the number of cases. The grouping criteria were as follows: ROP cases were included in the type 1 ROP group if both the eyes met the above criteria and received intravitreal injection of anti-VEGF drugs, or one eye met the criteria and received intravitreal injection while the contralateral eye with mild lesions did not receive treatment. ROP infants whose one eye or both eyes did not meet the criteria of type 1 ROP and received intravitreal injection were included in the nontype 1 ROP group. The curative effect, retinal pathological characteristics, and causes for the treatment of nontype 1 ROP were analyzed according to the number of eyes. The characteristics of included subjects were analyzed according to the number of cases.

### 2.4. Quality Assessment

The treatment effect was evaluated as follows: (1) Cured, complete retinal vascularization. Retinal vessels gradually grew to the ora serrata or less than 1 PD (papillary diameter) away from the ora serrata around zone III; (2) Improved, the retina was not completely vascularized. The retinal vessels had reached zone III, and there were still nonvascular areas, but without obvious active lesions at the last follow-up. (3) Recurrence. The tortuous dilation of retinal vessels was relived and the ridge became flattened in the early stage after the operation, but then the tortuous dilation of vessels, ridge aggravation, and neovascularization occurred again in the retina. (4) Aggravation. The tortuous dilation of retinal vessels was not significantly reduced, and the proliferation and traction were aggravated, even leading to retinal detachment. The improved cases still required regular examination. The recurrent cases were treated with intravitreal injection of anti-VEGF drugs again or retinal laser photocoagulation. The aggravating cases were treated with laser photocoagulation according to the retinal manifestations or surgery in case of retinal detachment.

The reasons for the treatment of nontype 1 ROP were as follows: (1) preplus diseases in the retina; (2) ridge aggravation, shown as more obvious ridges or ridge extension, or locally thickened and widened ridges, with a risk of increased proliferation; (3) simultaneous treatment of the contralateral eye; (4) no regression of lesions in the persistent stage 2 or 3 for over PMA (postmenstrual age) 44 weeks of follow-up; (5) logistical considerations, follow-up might not be timely due to various reasons (such as living far away, parents' poor understanding, and epidemic control policy).

### 2.5. Data Analyses

Data analyses were performed using the SPSS 19.0 statistical software. The differences in birth gestational age (GA), birth weight (BW), hospitalization days in NICU, first injection time, and follow-up time were compared by the *t*-test, and the differences in gender and drug types were compared by the *χ*^2^ test. The treatment effect was analyzed according to the number of afflicted eyes and compared by the *χ*^2^ test. *P* < 0.05 was considered statistically significant.

## 3. Results

### 3.1. Subject Characteristics

A total of 138 cases (262 eyes) of ROP, including 124 cases (89.9%) with both eyes and 14 cases (10.1%) with unilateral eyes, were included in the study. There were 99 cases (71.1%) in the type 1 ROP group and 39 cases (28.3%) in the nontype 1 ROP group, including 26 bilateral cases (18.8%) (52 eyes, 19.8%) and 13 unilateral cases (9.4%) (13 eyes, 5.0%). Of unilateral cases, 6 cases (4.3%) had type 1 ROP in one eye, and nontype 1 ROP in the contralateral eye.

The characteristics of subjects with the nontype 1 ROP and type 1 ROP are shown in Table 1. There was no significant difference in GA, BW, gender proportion, hospitalization days in NICU, and follow-up time between the two groups (*P* > 0.05). While, the time of the first treatment of the nontype 1 ROP group was slightly later than that of the type 1 ROP group (39.8 ± 2.7 weeks vs 38.1 ± 2.6 weeks, (*P* < 0.01). The difference in the types of anti-VEGF drugs was significant (*P* < 0.05), with a higher proportion of fusion protein drugs in the nontype 1 ROP group (87.2%) than that in the type 1 ROP group (54.5%).

### 3.2. Effect Assessment

According to the number of eyes (*n* = 262), 65 eyes (24.8%) were nontype 1 ROP. After treatment, 27 eyes (41.5%) were cured and 38 eyes (58.5%) were improved. There was no recurrence and aggravation. There were 197 eyes of type 1 ROP. After treatment, 79 eyes were cured (40.1%), 100 eyes were improved (50.8%), and 18 eyes recurred (9.1%), without aggravation cases. The difference in treatment effects between the two groups was significant (*P* < 0.05Table 2). All 18 recurrent eyes in the type 1 ROP group received intravitreal injection of the same anti-VEGF drugs and then the retinal conditions improved.

### 3.3. Reasons for the Treatment

**Figure 1:**
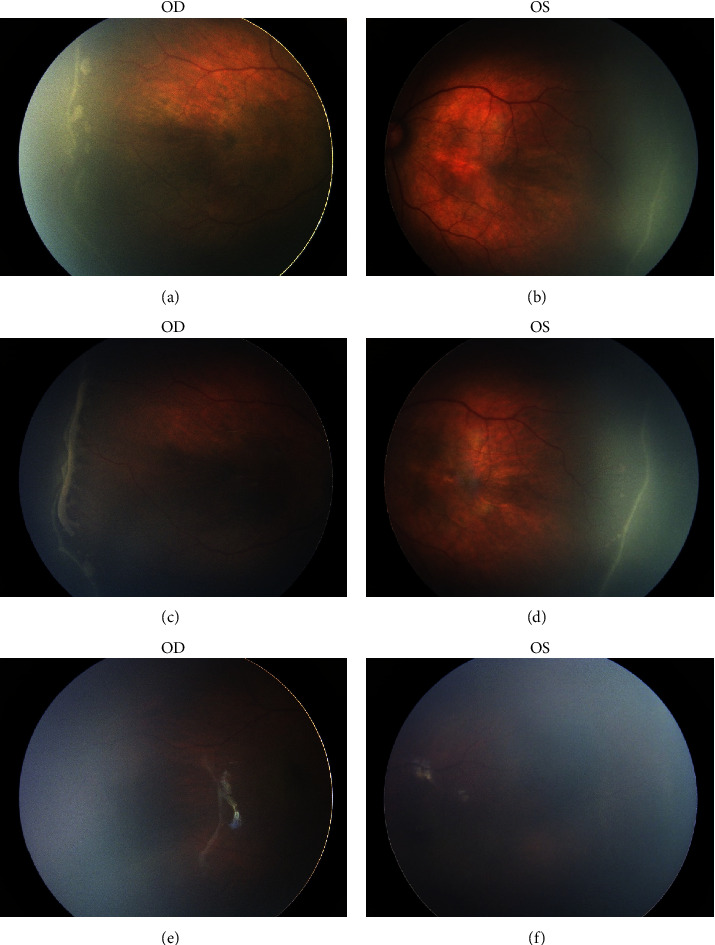
Nontype 1 ROP eyes considered to be treated due to multiple reasons. Case no. 3, male, GA 30 w, BW 1.3 kg. ROP in zone II, stage 2 with plus (−) in both the eyes was transferred to examination in PMA 39.1w (a, b). After 2 weeks (PMA 41.1w), the temporal ridge was thickened and widened with preplus in the right eye, which was ROP in zone II, stage 3 with preplus (c). As the contralateral eye, the ridge was more obvious and extended, which was still ROP in zone II, stage 2 with plus (−) (d). The bilateral retinal vessels grew to the periphery of zone III at 27.9 weeks (PMA 69.0w) after intravitreal injection of conbercept (e, f).

Of all treated eyes with nontype 1 ROP (*n* = 65) (Table 3), 63 eyes (96.9%) had ROP lesions in zone II, of which there were 5 eyes (3.1%) in posterior zone II and 2 eyes (3.1%) with ROP in zone I. In terms of stage, there were more lesions in stages 2 and 3, with 40 eyes (61.5%) and 23 eyes (35.4%), respectively. In terms of plus diseases, 58 eyes (89.2%) showed preplus. As for treatment reasons, the main reason was preplus in 58 eyes (89.2%), followed by ridge aggravation in 22 eyes (33.8%), simultaneous treatment in 9 eyes (13.8%) due to contralateral eye treatment, no regression of lesions in stage 2 or 3 for over PMA 44 weeks of follow-up in 8 eyes (12.3%), and logistical considerations in 4 eyes (6.2%). The above reasons could exist simultaneously (Figure 1). The preoperative features and specific reasons for treated nontype 1 ROP cases are shown in Table 4.

## 4. Discussion

Some previous studies analyzed the treatment reasons of nonype 1 ROP (Table 5) [3, 5, 11, 12], but the main treatment method in these studies was laser photocoagulation. No literature had been retrieved to explore the effect of intravitreal injection of anti-VEGF drugs on the treatment of nontype 1 ROP currently. Laser photocoagulation and anti-VEGF therapy are the main options for ROP currently. Laser therapy led to permanent destruction of the peripheral retina, and peripheral retinal vessels continued to develop after anti-VEGF agents' treatment [13]. As to efficacy compared to laser therapy, anti-VEGF agents as primary treatments had potential advantages for the eyes with posterior ROP (zone I type 1 ROP and A-ROP), and for the eyes with zone II type 1 ROP, anti-VEGF agents therapy showed similar efficacy; however, there was a significantly higher rate of reactivation [14]. Laser-treated eyes had a greater trend to myopia and astigmatism than anti-VEGF therapy [14, 15]. For the above reasons, anti-VEGF therapy performed under topical anesthesia was preferred for treatment with ROP during our clinical practice, and laser therapy was the used option for ROP with the risk of obvious fibrosis or the recurrent eyes. In addition, laser coagulation for ROP needed to be operated under general anesthesia which most parents of neonates were reluctant to choose in China.

Of the 263 eyes treated in this study, 65 eyes (24.8%) were nontype 1 ROP, showing a higher proportion than that in previous studies (9.5–13.7%) [3, 5, 11]. Because of that, the treatment for ROP cases in this study was an intravitreal injection of anti-VEGF drugs, which are simpler operated and more minimally invasive than laser therapy [13]. In this study, lesions in all eyes with nontype 1 ROP were relieved after treatment, which was similar to the results of previous studies [11]. It was also found that the treatment effect of the nontype 1 ROP eyes was better than that of type 1 ROP eyes, which may mainly be related to the milder condition. In this study, the proportion of fusion protein drugs used was higher in the nontype 1 ROP group (87.2%) than that in the type 1 ROP group (54.5%). This might be another potential reason for the difference in treatment effects between the two groups. Some retrospective studies found that the recurrence rate of the ROP eyes treated with fusion protein drugs (conbercept or aflibercept) was lower than that of the ROP eyes treated with ranibizumab [16, 17]. But a multicentral prospective trial comparing clinical outcomes of conbercept vs ranibizumab treatment for ROP found there was no significant statistical difference in the recurrence rate between the two anti-VEGF agents [18]. It is still controversial whether there is a difference between the efficacy of ranibizumab and fusion protein drugs in the treatment of ROP. The different proportion of drug selection in our study was associated with the time to market in China.

Of the eyes with nontype 1 ROP treated in this study, 63 cases (96.9%) had more lesions in zone II, and 40 eyes (61.5%) and 23 eyes (35.4%) had more lesions in stages 2 and 3, respectively. The characteristics of pathological manifestation were similar to those of the finding of Gupta et al. [3], in which 11 eyes (84.6%) had lesions in zone II and 12 eyes (92.3%) had lesions in stages 2 and 3. While in the study of Liu T et al. [5], most eyes (66%) had preplus lesions in zone II stage 3. The above evidence suggests that ROP should be checked carefully about the changes of lesions at stage 2 or 3 in zone II.

The main treatment reason for the nontype 1 ROP eyes in this study was the preplus disease (89.2%), which was different from the previous studies [3, 5, 11]. In the study of Gupta et al. and Rajan et al., the most important treatment reason was structural changes in the fundus caused by the traction of the ridge (69.2% and 72.7%, respectively) [3, 11]. Preplus disease (33.3%) was the second reason in the study of Rajan et al. [11]. The major reason in Liu et al.'s study was the contralateral eye with type 1 ROP (43%), followed by stage 3 ROP with preplus (30%) [5]. As for preplus and plus disease, ICROP3 defined it as a continuous spectrum of retinal vascular changes from normal to preplus and finally to plus disease. Consistent judgments of different scholars are only in the normal and last plus stages [10]. This suggests a high possibility of clinical disagreement over preplus lesions, resulting in no typical plus lesions in some ROP eyes and a further risk of retinal traction with progressive worsening of the ridge. Due to the use of anti-VEGF drugs, we paid more attention to the judgment of preplus in the ROP examination.

The second cause of treatment in this study was ridge aggravation (33.8%), shown as more obvious or/and more extension, or thickened and widened locally. Actually, the ridge aggravation was often accompanied by preplus (Figure 1). Koucheki et al. confirmed that preplus was significantly correlated with increased ridges (≥2 continuous clock hours of the persistent stage 3 crossing the temporal horizontal midline) in the eyes with stage 3 ROP persisting ≥40 weeks of PMA [12]. The ridge aggravation in this study was slightly milder than the structural changes such as macular traction, retinal traction, or folds produced due to the tangential traction caused by the straightening of the temporal vessels in the fundus mentioned in previous studies [3, 5, 11]. Under these fibrosis conditions, intravitreal injection of anti-VEGF drugs may not be recommended because of the risk of aggravated traction [19, 20].

In this study, the simultaneous treatment of the contralateral eyes accounted for 13.8%. Most previous studies considered that acute ROP commonly occurs in both the eyes. For example, 79.1% of ROP infants have high-risk prethreshold disease in both the eyes at the time of enrollment in an ETROP study [1]. A study on telemedicine approaches to evaluating of acute-phase retinopathy of prematurity (e-ROP) found that 72.7% of infants had the same severity of ROP in both the eyes among ROP image sessions [21].

However, our study does not recommend arbitrary early treatment for nontype 1 ROP. Previous studies suggested that ROP with stage 3 can be treated when no regression is found after 41 weeks of PMA [3] or continuous 6 weeks of follow-up [5, 11]. In this study, the average time of the first treatment for the eyes with type 1 ROP was 38.1 weeks, while the follow-up of another 6 weeks was 44 weeks for the nontype I ROP eyes with some of the above particular retinal features. Meanwhile, due to the use of anti-VEGF drugs, in order to avoid obvious fibrosis, we paid more attention to the progression of lesions at stage 2 and stage 3. Therefore, our study considered a treatment for ROP infants with lesions at persistent stages 2 and 3 without regression at PMA 44 weeks or more and whether there were other retinal manifestations were also taken into consideration. In this study, 4 cases (8 eyes) (12.3%) were followed up for ≥ PMA 44 weeks and then received anti-VEGF treatment, and the fundus was simultaneous with preplus lesions or ridge aggravation or logistical considerations before treatment. During the struggling follow-up period, more attention should be paid to the changes of extraretinal neovascular proliferation, and the anti-VEGF therapy should be performed in time before the fibrosis is obvious. Once obvious fibrosis has formed, laser coagulation will be recommended because retinal traction may be aggravated after anti-VEGF treatment [19, 20]. Unlike other ocular neovascular conditions (e.g., wetAMD), in which VEGF is continually released, there is a single burst of VEGF that promotes neovascularization in ROP [22]. The delayed anti-VEGF therapy given at a period when VEGF levels are decreasing may promote fibrosis driven by transforming growth factor-*β* (TGF-*β*) and connective tissue growth factor (CTGF) [23–25]. Traction from fibrosis may cause retinal detachments.

In this study, 4 eyes (6.2%), 2 cases (Nos. 4 and 5, Table 4) were treated for the logistical considerations, that is, follow-up might not be timely due to various reasons. Their parents lived far away or affected by epidemic control reasons (all eyes accompanied by other reasons, such as preplus or follow-up time ≥ PMA44w) and might not be followed up in time, and treatment was chosen considering that the retinal lesions tended to aggravate at the same time. The logistical considerations of Liu et al.'s study were the difficulty in follow-up or general anesthesia for non-ROP surgery (3%) [5]. The intravitreal injections for ROP infants in our study were performed under topical anesthesia, and there was no treatment under general anesthesia due to other diseases. For some ROP cases with difficulty in follow-up, detailed communication with the parents before treatment was recommended to emphasize the importance of follow-up, especially after anti-VEGF drug treatment that requires longer follow-up. In fact, the 2 cases were followed up in our hospital within 4 weeks after treatment. Then, the following examinations from the 6th week were started in the local hospital and the regular examination results including some retinal images would be transmitted to our research group through WeChat or the network of telemedicine.

Our study had some limitations. First, to avoid medical disputes caused by delayed treatment during clinical practice, there was no control group set. So, it was unable to accurately judge the progression of nontype 1 ROP with aggravating tendency if not treated. The sample in the study group will gradually be increased to collect and follow up carefully some untreated cases with nontype 1 ROP so as to investigate reasonable methods in the future. Second, as for the systemic effect of anti-VEGF drugs on premature infants, previous studies have confirmed that the intravitreal injection of anti-VEGF drugs in the ROP eyes has a certain inhibitory effect on the level of VEGF in blood [26–29]. But different anti-VEGF drugs have different effects. There were no differences in plasma-free VEGF concentrations of ROP infants after bilateral intravitreal injection of ranibizumab from the RAINBOW Trial [30]. Cheng et al. found that the serum VEGF levels in ROP infants were suppressed for a short time after intravitreal injection of conbercept and returned to the preoperative level at 4 weeks [28]. Huang CY found that serum VEGF levels in type 1 ROP infants were suppressed for 3 months after treatment with aflibercept or bevacizumab, but the suppression of systemic VEGF was more pronounced in infants treated with bevacizumab than those treated with aflibercept [29]. The suppression of VEGF in blood may affect the development of important organs of premature infants. Considering these potential risks, we are very cautious in the treatment of ROP and do not arbitrarily expand the treatment indications. In clinical practice, we always follow the professional guidelines and recommendations. Type 1 ROP eyes need treatment, while type 2 ROP eyes need close observation. However, continuous observation or treatment of ROP infants with aggravating tendencies but not reaching the typical fundus manifestations of type 1 was confused and should be discussed carefully. At the meanwhile, we have explored a lower dose of anti-VEGF drug intravitreal injection for ROP to reduce the possible systemic adverse reactions (published in Chinese, http://www.coretina.com/article/10.3760/cma.j.cn511434-20200219-00066).

## 5. Conclusions

Nontype 1 ROP with some characteristics, such as preplus, ridge aggravation, treatment with contralateral eyes, no regression in the persistent stage 2 or 3 for follow-up ≥ PMA 44 weeks, and logistical considerations, can be considered carefully to receive intravitreal injection of anti-VEGF drugs based on current expert consensus or guidelines.

## Figures and Tables

**Table 1:** Characteristics of subjects with type 1 and nontype 1 ROP (*N*  = 138).

Parameters^*α*^	Nontype type I ROP group	Type 1 ROP group	Statistic value^*β*^	*P* ^*β*^
	(*N* = 39)	(*N* = 99)		
GA (week)	28.5 ± 1.9	28.9 ± 2.2	1.033	0.303
BW (kg)	1.16 ± 0.35	1.19 ± 0.37	0.504	0.615
Gender (male, N (%))	21 (53.8)	65 (65.7)	1.662	0.197
Hospitalization days in NICU (day)	56.3 ± 23.2	53.6 ± 28.8	0.521	0.603
Time of the first treatment (week)	39.8 ± 2.7	38.1 ± 2.6	3.461	0.001
Follow-up time (week)	29.5 ± 10.1	33.6 ± 15.4	1.537	0.127
Anti-VEGF drugs (*N* (%))				
Ranibizumab	5 (12.8)	45 (45.5)	12.896	0.000
Fusion proteins (conbercept and aflibercept)	34 (87.2)	54 (54.5)		

^*α*^GA, gestational age; BW, birth weight. ^*β*^Difference of gender was analyzed by the *χ*^2^ test, and the remaining parameters were tested using the *t*-test.

**Table 2:** Comparison of effects for the eyes with type 1 and nontype 1 ROP after intravitreal injection of anti-VEGF drugs (*N*  = 262).

	Nontype 1 ROP eyes	Type 1 ROP eyes	*χ* ^2^ value^*α*^	*P*
Cured	27 (41.5)	79 (40.1)	6.514	0.02
Improved	38 (58.5)	100 (50.8)		
Recurrence	0 (0.0)	18 (9.1)		
Total	65 (100)	197 (100)		

**Table 3:** Characteristics of pathological manifestation of nontype 1 ROP receiving treatment.

Characteristics		Nontype 1 treated eyes (*N* = 65)	
		*N*	%
Zone	I	2	3.1
	II	63	96.9
	Posterior II	5	7.7
	Periphery II	58	89.2

Stage	1	2	3.1
	2	40	61.5
	3	23	35.4

Plus disease^*α*^	−	7	10.8
	±	58	89.2

Reasons	Preplus	58	89.2
	Ridge aggravation	22	33.8
	Contralateral eye	9	13.8
	Follow-up time ≥ PMA44w	8	12.3
	Logistical considerations	4	6.2

^*α*^±, referred to preplus diseases.

**Table 4:** List of nontype 1 ROP cases that received intravitreal injection of anti-VEGF drugs (*N*  = 39).

ID.	Gender	GA (w)	BW (kg)	Eyes	Zone^а^	Stage	Plus^*β*^	Range (hr)	Time of the first treatment (week)	Medicine^*β*^	Reasons for treatment^*γ*^
1	Male	26.6	0.95	OD	II	2	±	7	42.2	C	Preplus
				OS	II	2	±	7	42.2	C	Preplus
2	Male	27.6	1.00	OD	II	2	±	6	43.6	C	Follow-up time ≥44 w, ridge aggravation, preplus
				OS	II	2	±	6	43.6	C	Follow-up time ≥44 w, preplus
3	Male	30.0	1.30	OD	II	3	±	6	41.3	C	Ridge aggravation, preplus
				OS	II	2	−	6	42.3	C	Ridge aggravation, contralateral eye
4	Female	29.0	0.90	OD	II	2	−	5	45.7	C	Follow-up time ≥44 w, logistical considerations
				OS	II	2	-	5	46.7	C	Follow-up time ≥44 w, logistical considerations
5	Male	28.7	1.30	OD	I	1	±	12	34.0	C	Preplus, logistical considerations
				OS	I	1	±	12	34.0	C	Preplus, logistical considerations
6	Female	30.0	1.66	OD	II	2	±	5	38.0	R	Preplus
				OS	II	3	±	5	39.0	R	Preplus
7	Female	27.6	1.10	OD	II	3	±	12	38.6	R	Ridge aggravation, preplus
				OS	II	3	±	12	38.6	R	Ridge aggravation, preplus
8	Female	27.0	0.98	OD	II	2	±	5	36.3	C	Preplus
				OS	II	2	±	5	37.3	C	Preplus
9	Male	29.4	1.10	OD	II	2	±	6	40.7	R	Preplus
				OS	II	2	±	6	40.7	R	Preplus
10	Male	32.0	1.50	OD	II	3	−	6	40.9	R	Type 1
				OS	II	3	±	6	40.9	R	Ridge aggravation, preplus, contralateral eye
11	Male	30.7	1.66	OD	II	2	±	5	35.6	C	Preplus
				OS	II	2	±	7	35.6	C	Preplus
12	Male	30.0	1.63	OD	II	3	−	5	38.4	A	Type 1
				OS	II	3	±	5	38.4	A	Ridge aggravation, preplus, contralateral eye
13	Male	26.9	0.72	OD	II	3	±	12	37.6	C	Preplus
				OS	II	2	−	12	NA	NA	Untreated
14	Female	28.4	1.05	OD	II	3	−	5	44.7	C	Follow-up time ≥44 w, ridge aggravation
				OS	II	3	−	5	44.7	C	Follow-up time ≥44 w, ridge aggravation
15	Female	28.9	1.13	OD	Posterior II	2	−	12	39.8	C	Contralateral eye
				OS	Posterior II	2	+	12	39.8	C	Type 1
16	Female	27.0	0.73	OD	III	2	−	3	NA	NA	Untreated
				OS	II	3	±	4	39.7	C	Ridge aggravation, preplus
17	Female	26.7	0.71	OD	II	2	±	6	39.8	C	Preplus
				OS	II	2	±	6	39.8	C	Preplus
18	Male	27.3	1.10	OD	II	2	±	10	37.7	C	Preplus
				OS	II	2	±	10	37.7	C	Preplus
19	Female	25.6	0.90	OD	II	2	−	3	NA	NA	Untreated
				OS	II	3	±	3	39.6	C	Ridge aggravation, preplus
20	Male	26.7	0.85	OD	II	3	±	7	43.3	C	Ridge aggravation, preplus
				OS	II	2	−	6	NA	NA	Untreated
21	Female	25.9	0.86	OD	Posterior II	2	±	10	38.0	C	Preplus
				OS	Posterior II	2	±	10	38.0	C	Preplus
22	Male	26.9	0.68	OD	II	2	±	12	37.2	A	Preplus
				OS	II	2	±	12	37.2	A	Preplus
23	Male	27.6	1.10	OD	Posterior II	3	±	12	36.6	A	Preplus
				OS	Posterior II	3	±	12	36.6	A	Preplus
24	Male	28.0	1.17	OD	II	2	±	10	37.1	C	Preplus
				OS	II	2	±	10	37.1	C	Preplus
25	Male	31.0	1.75	OD	II	3	±	5	38.9	A	Ridge aggravation, preplus
				OS	II	2	−	5	38.9	A	Contralateral eye
26	Female	27.9	1.13	OD	II	2	±	5	40.5	C	Preplus
				OS	II	2	±	5	40.5	C	Preplus
27	Female	29.4	1.00	OD	II	2	±	6	43.4	C	Preplus
				OS	II	2	−	5	NA	NA	Untreated
28	Female	27.0	1.10	OD	II	3	±	5	42.6	C	Ridge aggravation, preplus
				OS	II	3	±	5	42.6	C	Ridge aggravation, preplus
29	Female	27.3	1.08	OD	II	2	±	5	37.7	C	Preplus
				OS	II	2	±	5	37.7	C	Preplus
30	Male	28.4	1.19	OD	II	2	−	4	NA	NA	Untreated
				OS	II	3	±	4	43.8	C	Preplus
31	Male	26.7	0.75	OD	II	2	±	10	40.8	C	Preplus
				OS	II	2	±	10	40.8	C	Preplus
32	Female	29.7	1.25	OD	II	3	±	6	40.0	C	Ridge aggravation, preplus
				OS	II	3	±	6	40.0	C	Ridge aggravation, preplus
33	Female	33.1	1.80	OD	II	2	±	5	44.8	C	Ridge aggravation, preplus, follow-up time ≥44 w
				OS	II	3	±	6	44.8	C	Ridge aggravation, preplus, follow-up time ≥44 w
34	Male	27.9	0.97	OD	II	2	+	12	36.9	R	Type 1
				OS	II	2	±	12	36.9	R	Preplus, contralateral eye
35	Female	27.9	1.18	OD	II	2	−	3	NA	NA	Untreated
				OS	II	3	±	3	39.8	C	Ridge aggravation, preplus
36	Male	31.9	2.28	OD	II	2	±	6	39.0	A	Contralateral eye, preplus
				OS	II	3	±	6	39.0	A	Ridge aggravation, preplus
37	Male	25.6	0.74	OD	II	2	±	12	39.0	C	Preplus
				OS	II	2	±	12	39.0	C	Preplus
38	Male	30.6	1.50	OD	II	2	±	6	37.7	A	Preplus, contralateral eye
				OS	II	2	+	6	37.7	A	Type 1
39	Female	29.0	1.26	OD	II	3	+	5	40.9	C	Type 1
				OS	II	2	±	6	40.9	C	Ridge aggravation, preplus, contralateral eye

^*α*^II referred to periphery zone II. ^*β*^ ± referred to preplus. ^*γ*^ R, C, and A represent ranibizumab, conbercept, and aflibercept. NA refers untreated. ^*δ*^One eye of type 1 ROP and the contralateral eye of nontype 1 ROP in 6 cases (ID. 10, 12, 15, 34, 38, 39). One eye of nontype 1 ROP and contralateral eye untreated in 7 cases (ID. 13, 16, 19, 20, 27, 30, 35).

**Table 5:** Previous studies of nontype 1 ROP treatment.

Investigator	Time published	Country	Method	Cases	Treatment	Reasons of treatment^*β*^
Gupta et al. [3]	2016	USA	Multicenter retrospective study	A total of 137 eyes treated, 13 eyes with nontype 1 ROP	Laser	Concerning structural changes, persistent ROP at an advanced PMA (41 w), vitreous hemorrhage, and active ROP with the fellow eye being treated for type 1 ROP
Liu et al. [5]	2019	USA	Multicenter retrospective study	A total of 1004 eyes, 126 eyes with nontype 1 ROP	Laser in 122 eyes and IVR^*α*^ in 4 eyes	Fellow eye with type 1 ROP, stage 3 ROP with preplus, and others: concerning structural changes in the retina; persistent stage 3 for ≥6 weeks without regression; stage 3 with no plus; stage 3, zone III with plus; logistical considerations; stage 2 disease.
Rajan et al. [11]	2020	India	Retrospective study	A total of 241 eyes treated, 33 eyes with nontype 1 ROP	Laser in 32 eyes, IVR^*α*^ in 1 eye	Structural changes, preplus disease, persistent stage 3 ROP that did not show any sign of regression for 6 weeks, and active ROP with the fellow eye being treated.
Koucheki et al. [12]	2020	Canada	Retrospective study	2,356 cases and 115 cases (172 eyes) with stage 3 ROP persisting ≥ PMA 40 w	Of 21 cases (33 eyes) treated by laser, 17 eyes with nontype 1 ROP	≥2 continuous clock hours of persistent stage 3 crossing the temporal horizontal midline and preplus

^*α*^IVR, intravitreal injection of bevacizumab. ^*β*^Reasons of treatment were arranged in the descending order of proportion.

## Data Availability

The data used to support the findings of this study are available from the corresponding author upon request.
